# Factors affecting survival after concurrent chemoradiation therapy for advanced hepatocellular carcinoma: a retrospective study

**DOI:** 10.1186/s13014-017-0873-1

**Published:** 2017-08-15

**Authors:** Ja Kyung Kim, Jun Won Kim, Ik Jae Lee, Seung-Moon Joo, Kwang-Hun Lee, Eun-Suk Cho, Jeong-Sik Yu, Tae Joo Jeon, Yonsoo Kim, Jung Il Lee, Kwan Sik Lee

**Affiliations:** 1 Department of Internal Medicine, Gangnam Severance Hospital, Yonsei University College of Medicine, 211 Eonjuro, Gangnamgu, Seoul, 06723 Korea; 2 Department of Radiation Oncology, Yonsei University College of Medicine, Seoul, Korea; 3 Department of Radiology, Yonsei University College of Medicine, Seoul, Korea; 4 Department of Nuclear Medicine, Yonsei University College of Medicine, Seoul, Korea; 5 Liver Cancer Clinic, Gangnam Severance Hospital, Seoul, Korea

**Keywords:** Hepatocellular carcinoma, Radiotherapy, Chemoradiotherapy, Chemoembolization

## Abstract

**Background:**

Concurrent chemoradiation therapy (CCRT) followed by hepatic arterial infusional chemotherapy (HAIC) was reported to be effective for advanced hepatocellular carcinoma (HCC) with portal vein thrombosis. However, transarterial chemoembolization (TACE) is not preferred in this setting. The aim of this study was to assess the factors affecting survival after CCRT, including additional TACE during repeated HAIC.

**Methods:**

Thirty-eight patients who underwent CCRT as the initial treatment for Barcelona Clinic Liver Cancer stage C HCC with vascular invasion between 2009 and 2016 were reviewed retrospectively. During CCRT, 5-fluorouracil (5-FU) was infused via chemoport during the first and last five days of five weeks of external beam radiation therapy. After CCRT, repeated HAIC with cisplatin and 5-FU was performed monthly. Nineteen patients (50%) underwent additional TACE between repeated HAICs. Factors related to overall survival and progression free survival (PFS) were analyzed.

**Results:**

The mean age of patients was 55 years (male:female, 33:5). Underlying liver diseases were hepatitis B, hepatitis C and non-B/C in 29, 1 and 8 patients, respectively. The median radiation dose was 4500 cGy. The objective response (OR) rate at one months after CCRT was 36.8%. The median PFS was 7.4 (range, 1.8 − 32.1) months. The median overall survival was 11.6 (range 2.8-65.7) months. Achieving an OR after CCRT (hazard ratio [HR], 0.028; *P* < 0.001), additional TACE (HR, 0.134, *P* < 0.001), and further rounds of HAIC (HR, 0.742, *P* = 0.001) were independent significant factors related to overall survival. The overall survival duration of patients with an OR after CCRT (median 44.2 vs. 6.6 months, *P* < 0.001) and additional TACE (median 19.8 vs. 9.1 months, *P* = 0.001) were significantly greater than those without an OR after CCRT or additional TACE.

**Conclusion:**

Patients who achieved an OR after CCRT, underwent additional TACE, and were subjected to repeated rounds of HAIC following CCRT showed better survival after CCRT for advanced stage of HCC with vascular invasion. A further prospective study is needed to confirm the positive effect of additional TACE after CCRT.

**Electronic supplementary material:**

The online version of this article (doi:10.1186/s13014-017-0873-1) contains supplementary material, which is available to authorized users.

## Background

Hepatocellular carcinoma (HCC) is the seventh-most common cancer in females and the fifth-most common cancer in males worldwide [1]. It is the second-most common cause of cancer-related deaths in males worldwide, with more than 500,000 deaths reported annually [2]. Viral hepatitis is the main cause of HCC, and endemic areas of viral hepatitis have a huge burden of this disease.

Since HCC is mostly asymptomatic, it is often detected at an advanced stage. Approximately one third of patients with HCC show macrovascular invasion, including portal vein thrombosis, on radiologic imaging [3]. Portal vein invasion of HCC is considered to represent aggressive tumor behavior and contribute to a higher risk of mortality [4]. According to the Barcelona Clinic Liver Cancer (BCLC) staging system, which is endorsed by the American Association for the Study of Liver Diseases and the European Association for the Study of the Liver, portal vein invasion of HCC is designated as advanced-stage HCC, and sorafenib is the recommended standard of care [5]. However, the Sorafenib Hepatocellular Carcinoma Assessment Randomized Protocol (SHARP) and Asia-Pacific SHARP trial showed a modest survival gain with sorafenib [6, 7]; therefore, new treatment strategies for portal vein invasion of HCC are required.

The Korean Liver Cancer Study Group (KLCSG) and the National Cancer Center (NCC) Korea practice guidelines and the Asia-Pacific Primary Liver Cancer Expert consortium recommend radiotherapy as an effective treatment for advanced HCC with portal vein invasion [8, 9]. Because radiotherapeutic strategies are frequently used in Korea, radiation therapy (RT) is suggested as an alternative treatment if sorafenib is not feasible [10]. The protocol of concurrent chemoradiation therapy (CCRT) followed by repeated hepatic arterial infusion chemotherapy (HAIC) was first developed for, and introduced in, patients with locally advanced HCC with portal vein invasion [11]. The CCRT protocol was developed before the era of sorafenib [11], when HAIC, transarterial chemoembolization (TACE), and RT were used to treat of advanced-stage HCC in Asian countries. Therefore, to increase the therapeutic effect, HAIC was added to deliver chemotherapeutic agents to HCC after CCRT. HAIC delivers chemotherapeutic agents at high concentrations through a chemoport. This results in the drug going through first-pass metabolism in the liver, which reduces the systemic level of the drug and decreases its systemic toxicity and side effects [12]. According to the previous report of favorable results [13, 14], HAIC was added to the protocol: HAIC was combined with 5-FU during RT for radiosensitizing effect, and HAIC was repeated monthly after CCRT to mainten the antitumor effect. The treatment showed a favorable objective response (OR) in 18 of 40 patients (45%), with a median survival duration of 13.1 months [11]. An updated outcomes report indicated that the median survival duration of 101 patients was 16.7 months. In the original protocol, HAIC was performed after CCRT, and HAIC was to be continued until a complete response was achieved. However, we occasionally experienced a decreased effect of repeated HAIC, which necessitated additional treatment for patients undergoing CCRT and repeated HAIC.

The use of TACE in patients with advanced HCC with major portal vein invasion is limited due to the possibility of liver failure and an increased risk of post-treatment complications [15]. The effect of TACE is also limited in these patients; multiple sessions of TACE or additive treatments are usually required. Therefore, TACE is generally not recommended in these patients. However, several studies have shown that TACE can be performed safely with no increase in morbidity or mortality [16–18]. Therefore, we tevaluated the efficacy of additional TACE between repeated HAIC after CCRT, which, to out knowledge, has not been assessed previously.

This study evaluated the factors affecting survival after CCRT, including additional TACE during repeated HAIC, in patients with advanced-stage HCC.

## Methods

### Patients

CCRT was performed in cases of advanced HCC with vascular invasion and reserved hepatic function, as decided by an institutional multidisciplinary HCC tumor board. We retrospectively analyzed patients who were diagnosed with advanced-stage HCC per the BCLC staging system and who underwent CCRT as the initial treatment between 2009 and 2016. We excluded patients who had intermediate-stage HCC (*n* = 13), a Child-Pugh score ≥ 9, Eastern Cooperative Oncology Group (ECOG) performance status >1, active peptic ulcer, TACE prior to CCRT (*n* = 5), no radiologic evaluation after CCRT, bile duct invasion (*n* = 2), a failed lesion after previous treatment (*n* = 2), other malignancy and inadequate hepatorenal function. Finally, we identified 60 patients who underwent CCRT, of whom 38 were enrolled in the study.

The institutional review board waived the requirement for obtaining informed consent for this retrospective study (No. 3-2017-0164). This study was conducted in compliance with the Declaration of Helsinki. All authors had access to the study data and have reviewed and approved the final manuscript.

### Diagnosis and definitions

The diagnosis of HCC was established by histological confirmation, typical dynamic radiologic findings on computed tomography (CT) and magnetic resonance imaging (MRI), or dynamic imaging with an elevated level of serum alpha-fetoprotein (AFP; > 200 ng/mL) or des-carboxy prothrombin (DCP; > 40 mAU/mL) according to the KLCSG-NCC Korea guidelines [8]. Portal/hepatic vein invasion was defined by the presence of an adjacent thrombus in the portal/hepatic vein confirmed by at least one imaging modality.

### CCRT followed by HAIC with/without TACE

CT-based three-dimensional treatment planning was performed in all patients. The gross tumor volume (GTV) was defined as radiographically abnormal areas detected in dynamic liver CT or MRI images. A margin of 0.5–1 cm in all directions was added to form the clinical target volume (CTV). Cranio-caudal movement of the liver determined from the range of diaphragmatic movement measured via fluoroscope or from all respiratory-motion phases via 4D CT were incorporated into the definition of the internal target volume (ITV). An additional 5 mm (to account for set-up error) was added to the ITV to define the planning target volume (PTV). Radiation was delivered using a linear accelerator (Elekta, Stockholm, Sweden) or Helical Tomotherapy (Accuray, Sunnyvale, CA, USA) with the intention to deliver 95% of the prescribed dose encompassing the PTV [19]. The total dose was determined by the fraction of the non-cancerous liver receiving 50% of the isocenter dose (V50%) according to the Yonsei University dose prescription guidelines. These guidelines were as follows: if <25% of the non-cancerous liver received 50% of the isocenter dose, the total dose should be increased to 59.4 Gy; if it was 25–50%, the dose should be reduced to 45–54 Gy; if it was 50–75%, the dose should be 30.6–41.4 Gy; and if it was > 75%, no treatment should be administered [20].

For concurrent chemotherapy, an indwelling chemoport was placed to deliver chemotherapeutic agents into the hepatic artery, along with RT. Subsequently, 5-FU (500 mg/day) was infused via the chemoport during the first and last five days of five weeks of external RT. For HAIC after CCRT, infusion of cisplatin (80 mg/m^2^ for 1 day) with 5-FU (750 mg/m^2^ for 3 days) through the chemoport was performed monthly [11]. In general, the decision to add TACE during repeated HAIC following CCRT was made in a multidisciplinary conference when tumor progression was suspected or a residual tumor persisted after repeated HAIC following CCRT. TACE was performed by infusion of approximately 10 mL of iodized oil (lipiodol; Guerbet, Aulnaysous-Bois, France) mixed with doxorubicin (50 mg) and selective embolization with gelatin sponge particles (Gelfoam; Upjohn, Kalamazoo, MI).

### Clinical data and response evaluation

Patients’ demographic and baseline clinical information—including cause of liver disease, tumor size, Child-Pugh score, ECOG performance status, and tumor marker levels—were collected. Four weeks after CCRT, dynamic imaging (CT or MRI) was performed to evaluate the response to CCRT. Treatment response was evaluated as complete response (CR), partial response (PR), stable disease (SD), or progressive disease (PD) using the modified response evaluation criteria for solid tumors [21]. After the first evaluation, follow-up tumor evaluation was repeated every 8 weeks. The dates of PD and death were recorded, and the progression-free survival (PFS) and overall survival (OS) durations were calculated from the start of CCRT. The OR was defined as the sum of PR and CR. Disease control was defined as SD, PR, or CR.

### Statistical analyses

Continuous variables are presented as means ± standard deviation or medians (range) and were compared by Student’s t-test or the Mann-Whitney test, as appropriate. Repeated measures (continuous variables) were compared by paired t-test or Wilcoxon signed-ranks test, as appropriate. Categorical variables were compared using Pearson’s chi-squared test. Local control, PFS, and OS rates were estimated by Kaplan-Meier analysis and compared using the log-rank test. Predictors of PFS and OS were identified by Cox regression analysis. Variables with *P* < 0.05 in the univariate analyses were entered into a Cox multivariate analysis. Hazard ratios and 95% confidence intervals were calculated. A *P*-value <0.05 was considered to indicate significance. To overcome the limitations imposed by the small sample size, backward-method Cox regression analyses were performed to identify minimum of clinically important variables using *P* < 0.05 for entry and *P* > 0.051 for removal in the stepwise procedure. All statistical tests were performed using PASW ver. 17.0 software (IBM Corp. Armonk, NY, USA).

## Results

### Patients’ characteristics

The baseline characteristics of the 38 patients enrolled in the study are shown in Table 1. The mean age was 55 years, and 86.8% of the patients were male. The most-common etiology of chronic liver disease was hepatitis B (76.3%). Liver cirrhosis was present in 30 patients (78.9%), and most patients (84.2%) were Child-Pugh class A. The mean tumor size was 11.5 cm. Main portal trunk invasion was noted in 16 patients (42.1%), and node metastasis was noted in 16 patients (42.1%). The mean and median serum AFP levels were 8460 and 1573 ng/mL, respectively. The mean and median serum DCP levels were 24,772 and 420 mAU/mL, respectively. The median radiation dose was 4500 cGy (range, 3060-6250 cGy).

**Table 1:** Baseline characteristics

Characteristics	*n* = 38
Age (years)	53 (34-77)
Male	33 (86.8)
Etiology	
HBV	29 (76.3)
HCV	1 (4.8)
NBNC	8 (18.9)
Liver cirrhosis	30 (78.9)
Child-Pugh class (A/B)	
Class A	32 (84.2)
Class B	6 (15.8)
ECOG performance status	
Grade 0	5 (13.2)
Grade 1	33 (86.8)
Vascular invasion	
Main portal trunk invasion	16 (42.1)
Left of right lobar portal vein invasion	15 (39.5)
Segmental portal vein invasion	5 (13.1)
Hepatic vein invasion	2 (5.3)
BCLC stage	
Advanced (C)	38 (100)
AJCC stage	
IIIB	20 (52.6)
IIIC	1 (2.6)
IVA	15 (39.5)
IVB	2 (5.3)
Tumor size (cm)	11.6 (3.5-21.1)
AFP (ng/mL)	1573.0 (2.0-54,000)
DCP (mAU/mL)	4378.0 (18.0-75,000)
Radiation dose (cGy)	4500 (3060-6250)
Round of HAIC	3.5 (0-18)
TACE	19 (50.0)
For local residual tumor	6 (31.6)
For newly recurring tumor	13 (68.4)

Variables are expressed as medians (range) or n (%)

*HBV* Hepatitis B virus, *HCV* Hepatitis C virus, *NBNC* Non-B and non-C, *ECOG* The Eastern Cooperative Oncology Group, *BCLC* Barcelona Clinic Liver Cancer, *AJCC* The American Joint Committee on Cancer, *AFP* α-fetoprotein, *DCP* Des-gamma carboxyprothrombin, *HAIC* Hepatic arterial infusional chemotherapy, *TACE* Transarterial chemoembolization

### Response after CCRT and decision to perform TACE

One month after CCRT, the mean tumor size decreased significantly from 11.5 cm to 8.6 cm (*P* < 0.001). Fourteen patients (36.8%) showed a PR, and six patients (15.8%) showed HCC progression (Table 2). The median duration of local control was 31 months, and the one-year local control rate of CCRT was 63.8% by Kaplan-Meier analysis. The median AFP level decreased from 1573 to 81 ng/mL (*P* = 0.002). Similarly, the median DCP level decreased from 4378 to 420 mAU/mL (*P* = 0.001). The OR rate was 36.8%, and the disease control rate was 84.2%.

**Table 2:** Response after concurrent chemoradiation therapy

Response	*n* = 38
Complete response	-
Partial response	14 (36.8)
Stable disease	18 (47.4)
Progressive disease	6 (15.8)
Objective response (CR + PR)	14 (36.8)
Disease control (CR + PR + SD)	32 (84.2)

Variables are expressed as n (%). *CR* Complete response, *PR* Partial response, *SD* Stable disease

TACE was added at least once during repeated HAIC in 19 patients (50%). There was no significant difference in the baseline characteristics between patients with and without additional TACE (Additional file 1: Table S1). Among the 19 TACE cases, six (31.6%) underwent TACE for residual tumor. In the remaining 13 cases (68.4%), TACE was performed to control recurrent lesions.

### Pfs

**Fig. 1:**
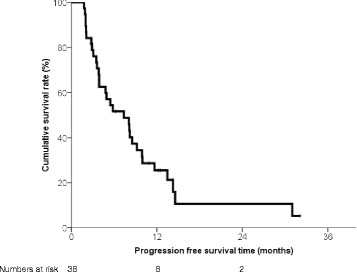
Progression-free survival (PFS) of the patients. The median PFS duration was 7.4 months

Twenty-nine patients (76.4%) showed HCC progression until the last follow-up visit (Table 2). First progression was noted in out-of-field lesions in most cases (27/29, 93.1%). The most-frequent site of out-of-field progressed lesions was the liver (15/27, 55.6%) (Table 3). The median PFS was 7.4 (range: 1.8 − 32.1, 95% CI: 3.7-11.0) months (Fig. 1). PFS was associated with the Child-Pugh class (*P* = 0.020), baseline DCP level (*P* = 0.005), and DCP level after CCRT (*P* < 0.001) in univariate analyses. Multivariate Cox regression analysis using the backward method revealed that DCP level after CCRT (*P* = 0.001) was an independent factor related to PFS (Table 4). The characteristics of patients with and without progression was compared. There was no significant difference (Additional file 1: Table S2.).

**Table 3:** Characteristics of disease progression

Progression		*n* = 38
No progression		9 (23.7)
Original lesion		2 (5.3)
New lesion		27 (71.1)
Liver	15 (55.6)	
Lung	10 (37.0)	
Bone	1 (3.7)	
Neck node	1 (3.7)	

Variables are expressed as n (%)

**Table 4:** Predictors of progression free survival

Characteristic	Univariate			Multivariate		
	Hazard ratio	95% CI	*P*-value	Hazard ratio	95% CI	*P*-value
Age (per year)	0.997	0.962-1.033	0.861			
Gender (male vs. female)	0.892	0.268-2.969	0.852			
Liver cirrhosis (with vs. without)	0.939	0.401-2.202	0.886			
Child-Pugh class (A vs. B)	0.304	0.111-0.829	0.020	0.461	0.155-1.366	0.162
Main portal trunk invasion (with vs. without)	1.098	0.520-2.318	0.805			
Node metastasis (with vs. without)	0.809	0.393-1.664	0.565			
Baseline tumor size (per 1 cm)	1.045	0.958-1.140	0.317			
Baseline AFP (per 100 ng/mL)	1.000	0.997-1.002	0.827			
Baseline DCP (per 100 mAU/mL)	1.002	1.001-1.003	0.005			
Radiation dose (per 100 cGy)	0.931	0.866-1.001	0.054			
Objective response after CCRT (with vs. without)	0.560	0.267-1.174	0.125			
AFP after CCRT (per 100 ng/mL)	1.000	0.998-1.002	0.962			
DCP after CCRT (per 100 mAU/mL)	1.004	1.002-1.006	<0.001	1.003	1.001-1.005	0.001
Additional TACE (with vs. without)	0.590	0.269-1.299	0.190			
Round of HAIC	0.914	0.831-1.005	0.062			

*AFP* α-fetoprotein, *DCP* Des-gamma carboxyprothrombin, *CCRT* Concurrent chemoradiation therapy, *TACE* Transarterial chemoembolization, *HAIC* Hepatic arterial infusional chemotherapy

### Os

**Fig. 2:**
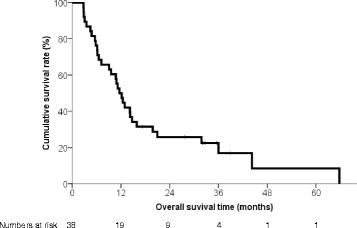
Overall survival of the patients. The median overall survival duration was 11.6 months

**Fig. 3:**
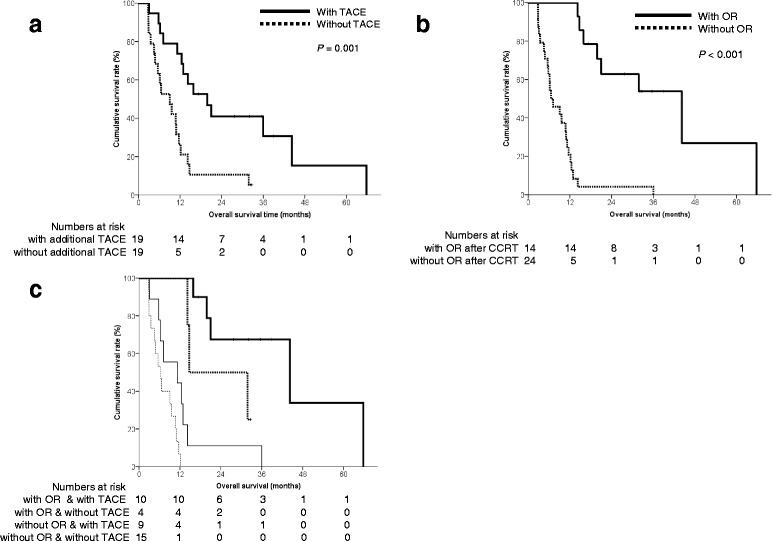
Kaplan-Meier survival curves according to (a) additional transarterial chemoembolization (TACE), (b) achievement of an objective response (OR) and (c) combination of additional TACE and/or achievement of an OR after CCRT

The median overall survival duration was 11.6 (range: 2.8-65.7, 95% CI: 9.267-13.999) months (Fig. 2). The characteristics of patients who survived and deceased were compared. Survived patients had no main portal trunk invasion, achieved ORs after CCRT, and less DCP levels after CCRT (Additional file 1: Table S3.). In univariate analyses, patients with Child-Pugh class A (*P* = 0.003), a PR after CCRT (*P <* 0.001), a lower DCP level after CCRT (*P* = 0.001), additional TACE (*P* = 0.001), and absence of main portal trunk invasion (*P* = 0.040) showed a better OS (Table 5). Among these factors, PR after CCRT (*P* < 0.001) and additional TACE (*P* = 0.002) were independently and significantly positively related to OS. The patients were categorized into four groups according to PR after CCRT and TACE;survival was significantly different among the four groups (log-rank test, *P* < 0.001; Fig. 3). Patients with PR after CCRT and additional TACE during repeated HAIC showed a greater median survival duration than patients with PR after CCRT and no additional TACE during repeated HAIC (44.2 vs. 14.7 months). Among patients who did not achieve a PR after CCRT, those who underwent additional TACE had a greater OS duration than those who did not (11.2 vs. 6.2 months).

**Table 5:** Predictors of overall survival

Characteristic	Univariate			Multivariate		
	Hazard ratio	95% CI	*P*-value	Hazard ratio	95% CI	*P*-value
Age (per year)	0.990	0.952-1.029	0.601			
Gender (male vs. female)	0.778	0.269-2.246	0.641			
Liver cirrhosis (with vs. without)	1.011	0.430-2.377	0.980			
Child-Pugh class (A vs.B)	0.245	0.090-0.668	0.003	0.436	0.155-1.226	0.116
Main portal trunk invasion (with vs. without)	2.079	1.019-4.243	0.040	2.099	0.941-4.682	0.070
Node metastasis (with vs. without)	0.732	0.350-1.531	0.405			
Baseline tumor size (per 1 cm)	0.912	0.830-1.002	0.056			
Baseline AFP (per 100 ng/mL)	1.000	0.998-1.003	0.711			
Baseline DCP (per 100 mAU/mL)	1.001	0.999-1.002	0.256			
Radiation dose (per 100 cGy)	1.007	0.943-1.074	0.843			
Objective response after CCRT (with vs. without)	0.096	0.036-0.254	<0.001	0.028	0.005-0.148	<0.001
AFP after CCRT (per 100 ng/mL)	1.000	0.998-1.003	0.702			
DCP after CCRT (per 100 mAU/mL)	1.003	1.001-1.004	0.001			
Additional TACE (with vs. without)	0.296	0.137-0.641	0.001	0.134	0.047-0.383	<0.001
Round of HAIC	0.805	0708-0.915	0.001	0.742	0.626-0.880	0.001

*AFP* α-fetoprotein, *DCP* Des-gamma carboxyprothrombin, *CCRT* Concurrent chemoradiation therapy, *TACE* Transarterial chemoembolization, *HAIC* Hepatic arterial infusional chemotherapy

## Discussion

Since the protocol of CCRT for advanced stage HCC with portal invasion was introduced [11], favorable results have been reported; e.g., a median survival duration of ≥11 months [19, 22–24]. In the current study, the median OS for CCRT was 11.6 months. By comparison, in the Asian-Pacific sorafenib trial, which included Korean patients, the median OS durations with and without sorafenib were 6.5 and 4.2 months, respectively [7]. Although these results cannot be compared directly, the overall outcomes of CCRT seem to be more favorable than systemic treatment without localized treatment.

Although sorafenib is the recommended first-line treatment for advanced stage HCC per the BCLC staging system [8], in practice, its use is limited by its high cost, reimbursement strategy, modest survival gain, and poor tolerance. In cases in whichthe use of sorafenib is not feasible, we apply radiation-based treatment using a median radiation dose of 45 Gy delivered in 25 fractions. Owing to the efficacy of RT, first progression occurred mainly due to development of new lesions outside of the radiation field.

During repeated HAIC, TACE was added in half of the cases in the current study. Although TACE is mainly recommended for the treatment of HCC without vascular invasion, it can also be applied to these patients [25]. Most primary lesions after CCRT remained stable and showed shrinkage of portal vein thrombosis. Thus, TACE can be used to treat such primary lesions with or without new lesions. Because TACE occludes tumor-supplying arteries, it may exert a more potent antitumor effect than HAIC.

PFS was related to the Child-Pugh class and baseline and post-CCRT DCP levels in univariate analyses. However, the DCP level after CCRT was the only independent predictor of PFS. DCP level has been reported as an effective tumor marker and is associated with large tumor size, vascular invasion, intrahepatic metastases, and a low grade of tumor cell differentiation [26]. A high preoperative DCP level appears to be indicative of recurrence in small HCC and facilitates detection of HCC recurrence after living-donor liver transplantation [27].

In this study, univariate analyses indicated that OS was related to the Child-Pugh class, main portal trunk invasion, achievement of an OR after CCRT, the DCP level after CCRT, further rounds of HAIC and additional TACE. However, achievement of an OR after CCRT, additional TACE, and further rounds of HAIC were independent factors associated with a good OS. Although OR after CCRT and further rounds of HAIC were fully predictable factors of improved survival, additional TACE between repeated HAIC after CCRT was, unexpectedly, an independent prognostic factor for improved survival. This retrospective study aimed to identify the factors affecting survival after CCRT to facilitate a future pilot trial of a modified protocol to improve outcomes. The original CCRT protocol included indefinite repetitions of HAIC after CCRT. Our results suggest that a prospective randomized controlled trial to assess the effect of additional TACE after CCRT is required.

An early AFP response and OR are reportedly independent factors associated with PFS and OS after repeated HAIC followed by CCRT [28]. However, this response can be measured only if a patient has an initially elevated AFP level, and cannot be manipulated for the modification of the protocol. However, we can consider additional TACE after CCRT to improve OS in practice.

This study has several limitations. First, the sample size was relatively small, and so the effect of additional TACE needs to be confirmed in a future larger-scale study, the sample size of which should be based on our findings. Second, because of the retrospective nature of this study, additional TACE procedures were performed at various time points. To overcome this limitation, future prospective studies should randomize the patients receiving TACE and consider the timing of additional TACE. Third, we could not perform a comparison of therapeutic efficacy with the recommended treatment, sorafenib. However, in a previous comparative study of CCRT and other treatments involving propensity score matching, CCRT showed a better OS than other treatments [23]. Also, sorafenib treatment for Child-Pugh class B patients was not eligible for national health insurance reimbursement.

## Conclusions

Patients who achieved an OR after CCRT, underwent additional TACE, and were subjected to further rounds of HAIC following CCRT showed better survival after CCRT for treatment of advanced-stage HCC with vascular invasion. Further prospective studies are required to confirm the positive effect of additional TACE after CCRT.

## Additional file

Additional file 1: Table S1. Baseline characteristics according to treatment after CCRT. **Table S2.** Baseline characteristics according to progression after CCRT. **Table S3.** Baseline characteristics according to survival. (DOCX 20 kb)

## Abbreviations

5-FU: 5-fluorouracil
AFP: Alpha-fetoprotein
AJCC: The American Joint Committee on Cancer
BCLC: Barcelona Clinic Liver Cancer
CCRT: Concurrent chemoradiation therapy
CR: Complete response
CT: Computed tomography
DCP: Des-carboxy prothrombin
ECOG: The Eastern Cooperative Oncology Group
HAIC: Hepatic arterial infusion chemotherapy
HCC: Hepatocellular carcinoma
MRI: Magnetic resonance imaging
OR: Objective response
OS: Overall survival
PD: Progressive disease
PFS: Progression free survival
PR: Partial response
RT: Radiation therapy
SD: Stable disease
SHARP: Sorafenib Hepatocellular Carcinoma Assessment Randomized Protocol
TACE: Transarterial chemoembolization
