# Supplementary material for: Factors affecting survival after concurrent chemoradiation therapy for advanced hepatocellular carcinoma: a retrospective study
Source: Radiat Oncol. 2017 Aug 15;12:133. doi: 10.1186/s13014-017-0873-1 (PMC5558652; doi:10.1186/s13014-017-0873-1)
Supplement: Additional file 1: Table S1. — Baseline characteristics according to treatment after CCRT. Table S2. Baseline characteristics according to progression after CCRT. Table S3. Baseline characteristics according to survival. (DOCX 20 kb) [file 13014_2017_873_MOESM1_ESM.docx]

Table S1. Baseline characteristics according to treatment after CCRT

| Characteristics | HAIC  (N=19) | HAIC + TACE  (N=19) | *P*-value |
| --- | --- | --- | --- |
| Age (year) | 57 ± 12 | 53 ± 9 | NS |
| Male | 17 (89.5) | 16 (84.2) | NS |
| Etiology |  |  | NS |
| HBV | 15 (78.9) | 14 (73.7) |  |
| HCV | 1 (5.3) | 0 (0) |  |
| Non-B, non-C | 3 (15.8) | 4 (26.3) |  |
| Liver cirrhosis | 17 (89.5) | 13 (68.4) | NS |
| Child-Pugh class (A/B) | 16/3 | 16/3 | NS |
| Main portal trunk invasion | 8 (42.1) | 8 (42.1) | NS |
| Node metastasis | 6 (31.6) | 10 (52.6) | NS |
| Tumor size (cm) | 10.732 ± 4.767 | 12.332 ± 3.451 | NS |
| AFP (ng/mL) | 277.3 (2.0~54,000) | 1682.1 (3.0~54,000) | NS |
| DCP (mAU/mL) | 6391.5 (18.0~75,000) | 2325.0 (19.0~75,000) | NS |
| Radiation dose (cGy) | 4886.8 ± 743.3 | 5184.2 ± 717.2 | NS |

Variables are expressed as mean ± standard deviation, median (range) or n (%).

HAIC: hepatic arterial infusional chemotherapy; TACE: transarterial chemoembolization; HBV: hepatitis B virus; HCV: hepatitis C virus; NBNC: non-B and non-C; AFP: α-fetoprotein; DCP; des-gamma carboxyprothrombin.

| Characteristics | With progression  (n=31) | Without progression  (n=7) | *P*-value |
| --- | --- | --- | --- |
| Age (year) | 56 ± 11 | 53 ± 13 | NS |
| Male | 28 (90.3) | 5 (71.4) | NS |
| Liver cirrhosis | 24 (77.4) | 6 (85.7) | NS |
| Child-Pugh class A | 25 (80.6) | 7 (100.0) | NS |
| Main portal trunk invasion | 12 (38.7) | 4 (57.1) | NS |
| Node metastasis | 14 (45.2) | 2 (28.6) | NS |
| Baseline tumor size (cm) | 11.9 ± 4.2 | 9.9 ± 3.9 | NS |
| Baseline AFP (ng/mL) | 8,202.7 ±15,937.3 | 11,226.8 ± 19,461.5 | NS |
| Baseline DCP (mAU/mL) | 27,428.9 ± 32,157.5 | 13,382.6 ± 27,412.6 | NS |
| Radiation dose (cGy) | 4,657.9 ± 618.2 | 4,809.3 ± 516.1 | NS |
| Objective response after CCRT | 12 (38.7) | 2 (28.6) | NS |
| AFP after CCRT (ng/mL) | 5,363.9 ± 14,035.7 | 7,828.4 ± 20,360.5 | NS |
| DCP after CCRT (mAU/mL) | 14,276.0 ± 24,862.5 | 5,973.1 ± 14,652.5 | NS |
| Additional TACE | 17 (54.8) | 2 (28.6) | NS |
| Round of HAIC | 4.3 ± 4.3 | 4.0 ± 4.3 | NS |

Table S2. Baseline characteristics according to progression after CCRT

AFP: α-fetoprotein; DCP: des-gamma carboxyprothrombin; CCRT: concurrent chemoradiation therapy; TACE: transarterial chemoembolization; HAIC: hepatic arterial infusional chemotherapy.

| Characteristics | With survival  (n=6) | Without survival  (n=32) | *P*-value |
| --- | --- | --- | --- |
| Age (year) | 54 ± 6 | 55 ± 12 | NS |
| Male | 5 (83.3) | 28 (87.5) | NS |
| Liver cirrhosis | 5 (83.3) | 25 (78.1) | NS |
| Child-Pugh class A | 6 (100.0) | 26 (81.3) | NS |
| Main portal trunk invasion | 0 (0) | 16 (100.0) | 0.030 |
| Node metastasis | 2 (33.3) | 14 (43.8) | NS |
| Baseline tumor size (cm) | 13.9 ± 4.3 | 11.1 ± 4.1 | NS |
| Baseline AFP (ng/mL) | 11,630.7 ±20,956.9 | 8,221.5 ± 15,750.1 | NS |
| Baseline DCP (mAU/mL) | 28,253.5 ± 25,609.1 | 24,097.6 ± 32,788.0 | NS |
| Radiation dose (cGy) | 4,853.3± 662.2 | 4,654.4 ± 590.3 | NS |
| Objective response after CCRT | 6 (100.0) | 8 (25.0) | 0.001 |
| AFP after CCRT (ng/mL) | 9,054.9 ± 22,018.8 | 5,206.0 ± 13,827.8 | NS |
| DCP after CCRT (mAU/mL) | 428.0 ± 793.2 | 15,081.4 ± 24,848.3 | 0.003 |
| Additional TACE | 5 (83.3) | 14 (43.8) | NS |
| Round of HAIC | 7.0 ± 5.9 | 3.8 ± 3.8 | NS |

Table S3. Baseline characteristics according to survival

AFP: α-fetoprotein; DCP: des-gamma carboxyprothrombin; CCRT: concurrent chemoradiation therapy; TACE: transarterial chemoembolization; HAIC: hepatic arterial infusional chemotherapy.
